# Free Tensor Fascia Lata Flap and Synthetic Mesh Reconstruction for Full-Thickness Chest Wall Defect

**DOI:** 10.1155/2013/914716

**Published:** 2013-09-28

**Authors:** Jumpei Ono, Akira Takeda, Minekatsu Akimoto, Akira Iyoda, Yoshio Matsui, Yukitoshi Satoh, Eiju Uchinuma

**Affiliations:** ^1^Department of Plastic and Aesthetic Surgery, Kitasato University School of Medicine, 1-15-1 Minami-ku, Sagamihara, Kanagawa 252-0344, Japan; ^2^Department of Thoracic Surgery, Kitasato University School of Medicine, 1-15-1 Minami-ku, Sagamihara, Kanagawa 252-0344, Japan

## Abstract

A large full-thickness chest wall defect over 10 cm in diameter requires skeletal reconstruction and soft tissue coverage. Use of various flaps for soft tissue coverage was previously reported, but en bloc resection in each case affects these flap pedicles and sizes. We present a case of a 74-year-old man with a soft tissue tumor involving the left lateral chest wall. We performed an en block resection and skeletal reconstruction using a mesh, free tensor fascia lata (TFL) flap for soft tissue coverage. This procedure could be performed in one position. A fixed fascia lata of the flap was also useful for tight reconstruction with the mesh. We suggest that free TFL and/or anterior lateral thigh flap is a useful technique to reconstruct anterior to posterior lateral chest wall defects.

## 1. Introduction

For a chest wall reconstruction, it is necessary to do a skeletal and soft tissue reconstruction. Management of the pleural cavity is important to decrease the rate of postoperative complications and mortality [1]. The availability of prosthetic materials influences the surgeon's choice, and complications are sometimes caused by those materials [2–4]. Using flaps to repair a full-thickness defect depends on the reconstruction portion and size of the chest wall defect. Full-thickness chest wall reconstruction may be performed with a myocutaneous flap such as the latissimus dorsi or rectus abdominis [5], but the pedicles of these flaps could be resected with the tumor in some cases. Here we report a full-thickness chest wall reconstruction by free tensor fascia lata (TFL) flap, using a fascia lata and mesh for skeletal support for a patient with malignant fibrous histiocytoma (MFH) involving the chest wall.

## 2. Case Report

**Figure 1:**
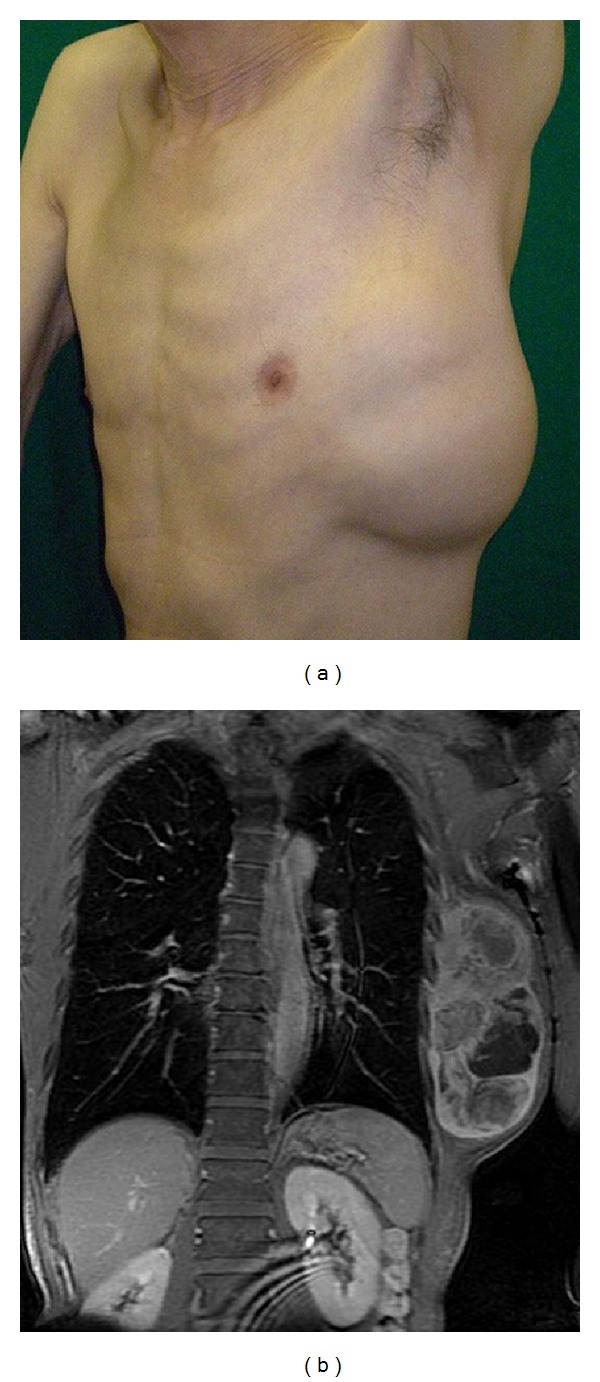
(a) Tumor of left lateral chest wall. (b) Preoperative magnetic resonance image of the tumor.

A 74-year-old, otherwise healthy, man presented with a rapidly enlarging mass in his left chest wall. On physical examination, a tumor of about 20 × 15 cm was detected (Figure 1(a)). Magnetic resonance imaging revealed a tumor mass with invasion of the latissimus dorsi and serratus anterior muscle but not the ribs (Figure 1(b)). Metastatic disease was ruled out on computed tomographic scans of the brain, chest, and abdomen. 

**Figure 2:**
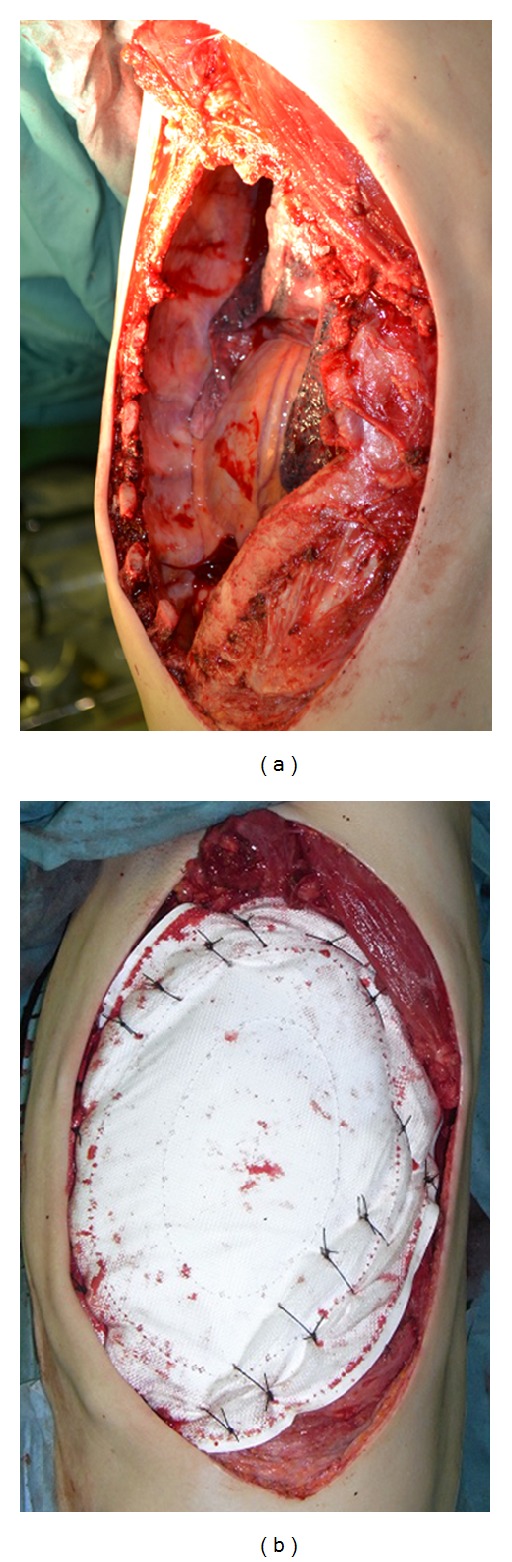
(a) After tumor resection. (b) Reconstructed chest wall by Composix E/X Mesh.

En bloc tumor and chest wall resection was performed with the patient in the right lateral position under the single-lung ventilation. The latissimus dorsi and serratus anterior muscles, the 4th through the 8th ribs, as well as those intercostal muscles with 3 cm margins from the tumor were resected through the marginal skin incision (Figure 2(a)). The pathological examination of the en bloc resected tumor revealed that it was approximately 30 × 13 cm. Finally, we reconstructed the chest wall defect using Composix E/X Mesh (Davol Inc., Bard Inc., USA) composed of expanded polytetrafluoroethylene (ePTFE) mesh and polypropylene mesh. We sutured the mesh to the ribs, from the 4th through to the 8th, and to these intercostal muscles outside the extra pleural cavity to provide stability (Figure 2(b)).

**Figure 3:**
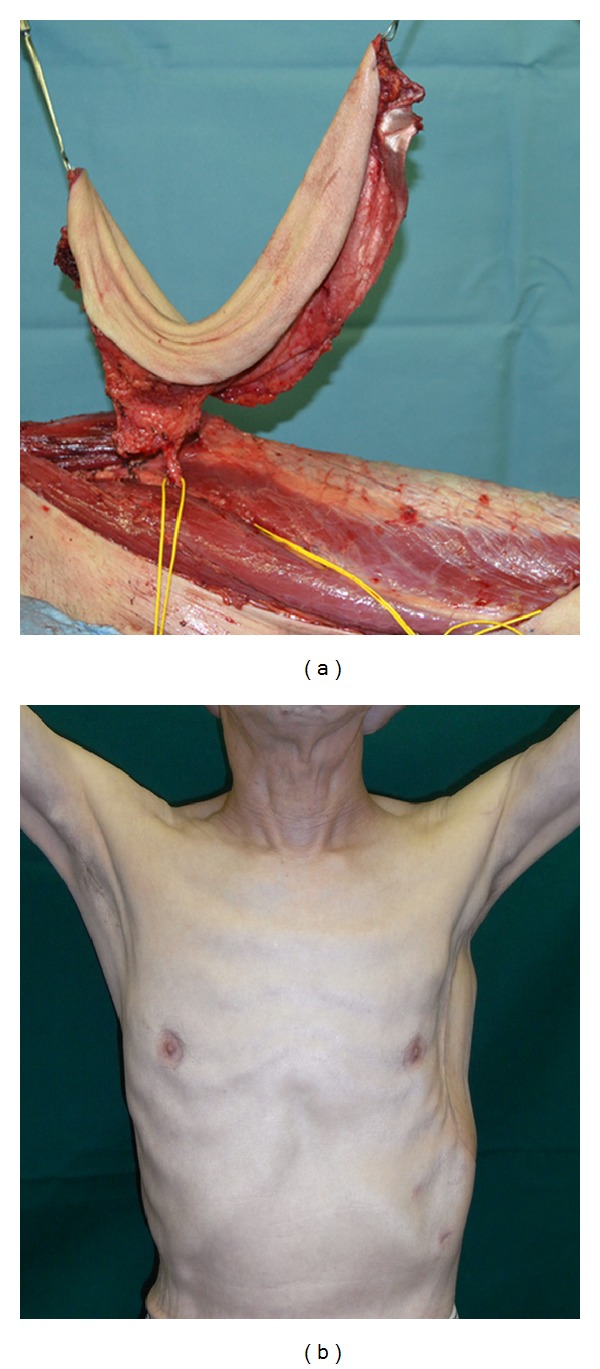
(a) A free tensor fascia lata flap was elevated in the ipsilateral thigh. (b) Six months after the chest wall reconstruction.

The free TFL flap was then elevated at the ipsilateral thigh, and the transverse branch of the lateral circumflex femoral vessels was dissected (Figure 3(a)). The thoracodorsal vessels and subcutaneous veins were used as the recipients for end-to-end anastomoses under loupe magnification. The fascia lata of the flap was sutured to the outside of the mesh to add more stability and strength. A chest tube was inserted into the thoracic cavity, and a drain was placed under the flap. Donor sites were grafted with a meshed split-thickness skin graft from the posterior thigh.

Histopathology showed a storiform arrangement of malignant cells, including osseous and cartilageinus tissue; these findings are comparable to those of storiform-pleomorphic MFH. 

The flap remained 100% viable with no evidence of skin necrosis. At a 6-month postoperative followup, the patient become well with no disfunction of breathing and disease free (Figure 3(b)).

## 3. Discussion

The selection of material for skeletal reconstruction and the flap for soft tissue coverage is of utmost importance in chest wall reconstruction. Perioperative complications can affect mortality. The selection of flap is influenced by the patient's intraoperative position and by the defect itself. Reconstruction using the rectus abdominis musculocutaneous (RAM) flap and the latissimus dorsi (LD) flap has been carried out for relatively large defects [4–6]. Sometimes these flap pedicles are excised during en bloc resection of tumor. In some cases of the lateral wall resection, thoracodorsal vessels, and latissimus dorsi muscle could be resected with tumor. Rectus abdominus myocutaneous flap is recommended in the lateral chest wall reconstruction as well as LD [6]. But rectus abdominus myocutaneous flap is bulky in most cases. A free TFL flap can be combined with an anterior lateral thigh (ALT) flap by using the lateral circumflex system of femoral vessels and can be of the same size as, or larger than, the RAM and LD flaps [7] reported for chest wall reconstruction [8]. 

In the present case, we planned to use a combined TFL/ALT flap at first but then, we chose a free TFL flap for a musculocutaneous perforator to shorten the operating time. For anterolateral, lateral, and posterolateral chest wall defect reconstructions, en bloc resection and reconstruction can be performed with the patient in one position by two teams. The thickness and roundness of the chest wall and that of the thigh are similar; therefore, it is simple to preoperatively determine the possibility of covering a chest wall defect using a thigh flap. We think that the free TFL and/or ALT flap is optimal in a lateral chest wall reconstruction. Free TFL flap was reported to back up procedure after latissimus dorsi flap and rectus abdominal flap necrosis in the chest wall reconstruction [9].

Skeletal reconstruction is necessary for the management of the pleural cavity. Prosthetic materials have been made of Prolene, Vicryl, ePTTE, Marex, and Goretex [4–6]. The surgeon's preference affects the selection of the prosthetic material [6]. In this case, we used Composix E/X mesh, which is a composite of ePTFE mesh and polypropylene mesh. Rib and omentum are useful as autologous tissues, but there is a limit to the amount of tissue that can be harvested from these, and the burden of the donor site is large [4–6]. Using the fascia lata for chest wall reconstruction only has been discussed [10]. The strength of the fascia lata reconstruction is weaker than that of using mesh; however, its strength can be increased by adding fascia lata on the outside of the mesh-skeletal reconstruction.

While the patient is in a single position for an anterior to posterior lateral chest wall reconstruction, free TFL and/or ALT flap reconstruction is an operation that can be more robust than that with a single mesh.

## 4. Conclusion

Free TFL and/or anterior lateral thigh flap is a useful technique to reconstruct anterior to posterior lateral chest wall defects.
